# Comparative experimental and theoretical study on anomalous Nernst effect of Heusler alloy Co_2_FeSi thin film: estimation of on-site Coulomb interaction at Co site

**DOI:** 10.1080/14686996.2025.2564061

**Published:** 2025-09-22

**Authors:** Weinan Zhou, Keisuke Masuda, Kazuki Sumida, Yuichi Fujita, Akio Kimura, Yuya Sakuraba

**Affiliations:** a Research Center for Magnetic and Spintronic Materials (CMSM), National Institute for Materials Science (NIMS), Tsukuba, Japan; b Research Institute for Synchrotron Radiation Science, Hiroshima University, Higashi-Hiroshima, Japan; c Graduate School of Advanced Science and Engineering, Hiroshima University, Higashi-Hiroshima, Japan; d International Institute for Sustainability with Knotted Chiral Meta Matter (WPI-SKCM^2^), Hiroshima University, Higashi-Hiroshima, Japan; e Synchrotron Radiation Research Center, National Institutes for Quantum Science and Technology (QST), Sayo, Japan

**Keywords:** Heusler alloy, Co_2_FeSi, on-site Coulomb interaction, anomalous Nernst effect, anomalous Nernst conductivity, anisotropic magnetoresistance effect, anomalous Hall effect, temperature dependence

## Abstract

Co_2_FeSi is considered a half-metallic ferromagnet and a Weyl semimetal, however, its predicted properties have been shown to be heavily influenced by how the on-site Coulomb interaction is incorporated, which remains controversial. In this study, we measure the anomalous Nernst conductivity (*α*_*xy*_) and anisotropic magnetoresistance (AMR) effect of a Co_2_FeSi thin film from low temperature to room temperature, and compare the results with those of first-principles calculations using different values for on-site Coulomb interaction at the Co site (*U*_Co_). Our measurements reveal that *α*_*xy*_ is less than 0.1 A m^−1^ K^−1^ at room temperature and decreases slightly with the temperature. The observed values of *α*_*xy*_ are more than one order of magnitude smaller than the predictions unless a small but finite *U*_Co_ is incorporated. The AMR effect exhibits a notable sign change from negative to positive with increasing temperature, which is inconsistent with the predicted band structure calculated using a large *U*_Co_ value. By combining these experimental observations with first-principles calculations, we estimate that the appropriate *U*_Co_ value is approximately 1–2 eV. Our findings provide valuable insight into the correlation effect in Co_2_FeSi, emphasizing the critical role of the on-site Coulomb interaction in accurately describing the transport properties of Co-based Heusler alloys.

## IMPACT STATEMENT

Experimentally determined anomalous Nernst conductivity of Co_2_FeSi agrees well with first-principles calculations when on-site Coulomb interaction at Co site is incorporated, thus provide valuable insights for materials exploration.

## GRAPHICAL ABSTRACT

**Figure:**
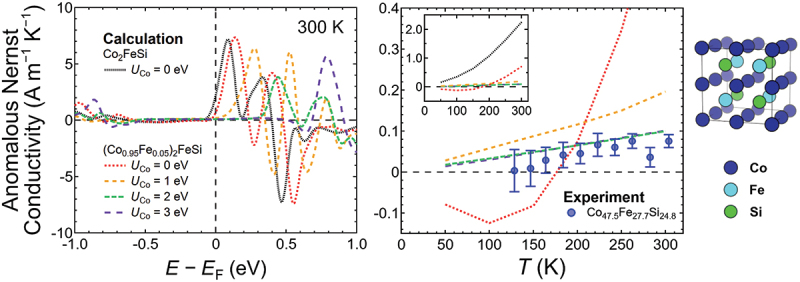

## 1. Introduction

Co-based Heusler alloys in the *L*2_1_ structure have attracted considerable interest in recent years because of their diverse and tunable properties. Many of these alloys are predicted to exhibit half-metallicity, *i.e*., a bandgap in one spin channel at the Fermi level (*E*_F_), resulting in 100% spin polarization [1–5]. This unique property significantly enhances magnetoresistance effects [6,7] and improves the efficiency of spin injection into nonmagnetic materials [8,9]. Exploiting this property has been a major approach for advancing the performance of spintronic devices such as magnetic sensors, magnetic random access memory (MRAM), nonlocal spin valves, and other related applications [10–12].

Meanwhile, it has been shown that some Co-based Heusler alloys can be classified as Weyl semimetals, possessing Weyl cones or nodal lines near *E*_F_ [13–17]. Such topologically nontrivial band structures induce large Berry curvature and are responsible for transport phenomena such as the large anomalous Hall effect (AHE) in Co_2_MnAl [18] and large anomalous Nernst effect (ANE) in Co_2_MnGa [17,19–21]. These features have inspired novel devices and functionalities leveraging these effects [10–12,22–24].

Among Heusler alloys, Co_2_FeSi is a well-known and extensively studied material. Experiments on bulk Co_2_FeSi have shown a notably high Curie temperature of approximately 1100 K and large magnetic moment of 6 *μ*_B_/f.u. [25], which agrees with the magnetic moment for a half-metallic full-Heusler alloy predicted by the Slater-Pauling rule [3]. By contrast, first-principles calculations predicted smaller magnetic moments of approximately 5 *μ*_B_/f.u. [25,26]. This discrepancy was addressed by incorporating the on-site Coulomb interaction (*U*) in calculations. When assuming *U* values of 2.5–5.0 eV for the Co site and 2.5–4.5 eV for the Fe site, calculations not only yield a magnetic moment that is consistent with the experimental value but also exhibit a clear half-metallic energy gap in the minority-spin channel; while calculations show that Co_2_FeSi is an ordinary ferromagnet rather than a half-metallic material if *U* is not taken into consideration [26,27]. However, experiments to determine the transport properties in relation to the half-metallicity of Co_2_FeSi have produced mixed results. Magnetotransport measurements on a single crystal showed evidence for half-metallicity, as electron-magnon scattering was exponentially suppressed at low temperatures owing to gapped spin-flip scattering [28]. However, the experimentally extracted energy separation between *E*_F_ and the conduction band (CB) edge of the minority spin was significantly smaller than that predicted theoretically. The anisotropic magnetoresistance (AMR) effect was employed to study the half-metallicity of Co_2_FeSi. According to the extended two-current model for AMR, which accounts for all *s-d* electron scattering processes through different spin channels, a half-metallic material is expected to consistently exhibit a negative AMR ratio [29]. Notably, other half-metallic candidates such as Co_2_MnSi and Co_2_FeGa_0.5_Ge_0.5_, whose half-metallic nature has been demonstrated by tunneling magnetoresistance (TMR) [6] or current-perpendicular-to-plane giant magnetoresistance (CPP-GMR) effect [7], have shown negative AMR ratios from low to room temperature, with minimal temperature dependence [30,31]. By contrast, Co_2_FeSi exhibits a negative AMR ratio at low temperature and a positive ratio at room temperature, suggesting the collapse of its half-metallic nature as a result of thermal excitation [30]. Point-contact Andreev reflection spectroscopy measurements have indicated significantly lower spin polarization for Co_2_FeSi than expected [32–35]. Furthermore, TMR and nonlocal spin valve devices using Co_2_FeSi have shown results due to low spin polarization as well [36,37]. Consequently, the significance and necessity of incorporating *U* in describing Co_2_FeSi remain controversial. Previously, Sumida *et al*. carried out resonant photoelectron spectroscopy measurements on a Co_2_FeSi thin film and found that Fe 3*d* states existed approximately 2 and 4 eV below *E*_F_, which was consistent with the calculated results without considering *U* at the Fe site [38]. Nevertheless, the impact of introducing *U* at the Co site appeared to be too subtle to produce observable changes when the experimental and calculated results were compared.

In this study, we investigated the impact of incorporating *U* at the Co site by measuring the anomalous Nernst conductivity (*α*_*xy*_) of a Co_2_FeSi thin film. *α*_*xy*_ is closely linked to the Berry curvature of the electronic band structure and is particularly sensitive to the bands near *E*_F_, in contrast to the anomalous Hall conductivity (*σ*_*xy*_), which accounts for contributions from all occupied bands [15,39]. Therefore, *α*_*xy*_ has been used to study magnetic Weyl semimetals such as Co_2_MnGa [17,19–21] and Co_3_Sn_2_S_2_ [40–42], along with other magnetic materials with topologically nontrivial band structures [43–47]. For Co_2_FeSi, Noky et al. theoretically predicted large magnitude of *σ*_*xy*_ and *α*_*xy*_ slightly above *E*_F_ without considering *U*, and identified the source of Berry curvature to be Weyl point [48]. The band structure of Co_2_FeSi can be found in an earlier study by Huang et al. where the negative peak of *σ*_*xy*_ slightly above *E*_F_ can be attributable to multiple band crossings in the minority spin, mostly along the X – W direction [35]. The value of *α*_*xy*_ for Co_2_FeSi at *E*_F_ has been predicted to be 2.57 A m^−1^ K^−1^ [48]. This is comparable to that for Co_2_MnGa, which currently holds the record for the anomalous Nernst coefficient (*S*_ANE_) at room temperature [17,19–21]. However, no large *S*_ANE_ value for Co_2_FeSi has yet been reported. Our experimental measurements revealed a significantly smaller *α*_*xy*_ value of less than 0.1 A m^−1^ K^−1^, with small temperature dependence. By comparing these experimental findings with first-principles calculations employing various values for *U* at the Co site (*U*_Co_), we found good agreement between the experimental and theoretical results when a finite *U*_Co_ value larger than 1 eV was assumed. Additionally, we measured the temperature dependence of the AMR effect using the same Co_2_FeSi thin film and observed a sign change from negative to positive with increasing temperature. This behavior contradicted the calculated band structures when assuming a *U*_Co_ value larger than 2 eV, thus providing an upper boundary. Collectively, these experimental results indicate that a small but finite value of *U*_Co_ is most appropriate for accurately describing Co_2_FeSi.

## 2. Methods

The Co_2_FeSi thin film was epitaxially grown on an MgO (100) single-crystal substrate via magnetron sputtering. The base pressure of the sputtering chamber was< 3 × 10^−6^ Pa. Prior to deposition, the surface of the MgO substrate was cleaned by heating at 600°C for 30 min. Then, the Co_2_FeSi thin film was formed by the co-deposition of a Co_50_Fe_25_Si_25_ alloy target and pure Si target. During the deposition, 120 W of DC power was supplied to the Co_50_Fe_25_Si_25_ target, and 90 W of RF power was supplied to the Si target, under an Ar pressure of 0.9 Pa. The distance between the targets and substrate was ~25 cm. The deposition was carried out for 27 min while maintaining the substrate temperature at 600°C. After the co-deposition, the sample was cooled to room temperature, followed by the deposition of a 2-nm-thick Al capping layer to prevent oxidation. To determine the composition, we used a quantitative application of an X-ray fluorescence (XRF; ZSX Primus II, Rigaku, Japan) analysis system, which was created by measuring multiple reference samples prepared in the laboratory. The compositions of these reference samples were determined using inductively coupled plasma mass spectroscopy. The composition and thickness of the Co_2_FeSi were Co_47.5_Fe_27.7_Si_24.8_ and 41.7 nm, respectively. The deposition rate was estimated to be 0.26 Å s^−1^. The thin film structure was studied using X-ray diffraction (XRD; SmartLab, Rigaku, Japan) with Cu *K*_*α*_ radiation. The magnetic properties were studied using a superconducting quantum interference device. To characterize the transport properties, the Co_2_FeSi thin film was patterned into a 2-mm-wide and 8-mm-long Hall bar using photolithography and Ar-ion milling. Subsequently, a lift-off process was used to form Au electrodes to improve the electrical connection with the Co_2_FeSi. We also microfabricated on-chip thermometers consisting of 50-nm-thick Pt wires on the same substrate adjacent to the Co_2_FeSi thin film. (See the Supplemental Material for a photograph of the patterned sample and schematic representation of the experimental configuration.) A homemade holder was used to measure the Seebeck coefficient (*S*_SE_) and *S*_ANE_. The sample was bridged between a Cu heat sink and Cu block, which was thermally connected to a heater to generate a temperature gradient (∇*T*) along the thin-film plane. The holder was placed in a Physical Property Measurement System (PPMS VersaLab; Quantum Design) to control the temperature (*T*) and magnetic field (*H*). To accurately measure ∇*T*, the resistance of the on-chip thermometers was measured using a four-terminal method. A source meter applied 10 μA through two thermometers, while two nanovoltmeters measured the voltages to determine their resistance. Before applying power to the heater and creating ∇*T* along the thin-film plane, we calibrated the on-chip thermometers by measuring their resistance as a function of *T* under zero *H*. Then, during the measurements of the *H* dependence of the thermoelectric signals, we also measured the thermometers when *H* was zero. These resistance values were then converted to *T* values. The difference in the *T* values of the two thermometers was divided by their separation (4 mm) to obtain the value of ∇*T*, while their average was used as the temperature for the obtained *S*_ANE_ and *S*_SE_. This measurement configuration was used in a previous study [49]. The longitudinal and transverse resistivities of Co_2_FeSi were measured using four-terminal sensing with a standard resistivity puck while varying the value of *H* applied along the out-of-plane direction. The AMR of Co_2_FeSi was measured using four-terminal sensing with a rotator, where a *μ*_0_*H* value of 0.3 T was applied in the thin-film plane.

For first-principles calculations, we first obtained the electronic structure of *L*2_1_-ordered Co_2_FeSi on the basis of the density-functional theory, including the spin-orbit interaction, which was implemented in the Vienna *ab initio* simulation program (VASP) [50]. We adopted the generalized gradient approximation (GGA) [51] for the exchange-correlation energy and used the projected augmented wave (PAW) pseudopotential [52,53] to properly treat the effect of core electrons. The lattice constant of the cubic unit cell was fixed at 5.64 Å, as determined from the diffraction peak position observed in the XRD measurement. Because the previous experimental results agreed with the calculations without considering *U* at the Fe site [38], *U* was considered only for the Co site and was varied from 0 to 3 eV. Using the obtained electronic structure, we calculated *σ*_*xy*_ as follows [54]:

$$\sigma_{\mathrm{xy}}\left(\epsilon \right)=-\frac{e^{2}}{\hbar }\int \frac{d^{3}k}{{\left(2\pi \right)}^{3}}\Omega^{z}\left(k\right),$$

Ωzk=−ℏm2∑nfEn,k,ϵ∑n ′≠n2Im⟨ψn,kpxψn ′,k⟩⟨ψn ′,kpyψn,k⟩En ′,k−En,k2,

where $\Omega^{z}\left(k\right)$ is the Berry curvature. Here, $p_{x}$ ($p_{y}$) is the *x* (*y*) component of the momentum operator, *n* and $n{\;}^{\prime }$ are the band indices, $\psi_{n,k}$ is the eigenstate with eigenenergy $E_{n,k}$, and $f\left(E_{n,k},\epsilon \right)$ is the Fermi distribution function for band *n* and wave vector **k** at energy ε relative to *E*_F_. In the calculation of *σ*_*xy*_, the direction of the magnetization was set to be along the [001] direction and 91 × 91 × 91 *k* points were used for the Brillouin zone integration to ensure good convergence for *σ*_*xy*_. On the basis of the Boltzmann transport theory, we calculated the transverse thermoelectric conductivity, *α*_*xy*_, for a given temperature *T* by using the obtained *σ*_*xy*_ in the following expression:

$$\alpha_{\mathrm{xy}}=-\frac{1}{\mathrm{eT}}\int \mathrm{d\epsilon}\left(-\frac{\mathrm{\partial f}}{\partial \epsilon }\right)\left(\epsilon -\mu \right)\sigma_{\mathrm{xy}}\left(\epsilon \right),$$

where $f=1/\left[\exp\left(\left(\epsilon -\mu \right)/k_{B}T\right)+1\right]$ is the Fermi distribution function, with *μ* being the chemical potential. Here, μ=0 corresponds to *E*_F_. All of these calculations were also conducted for (Co_0.95_Fe_0.05_)_2_FeSi with the *L*2_1_ order to theoretically consider the effect of the off-stoichiometry of the prepared Co_2_FeSi thin film on *σ*_*xy*_ and *α*_*xy*_. This treatment is supported by a previous study on Co_2_FeGa_0.5_Ge_0.5_, which showed that the potential energy barrier for Fe atoms to occupy Co sites and form Co-Fe disorder is lower than that for Ga or Ge [55]. Here, we used the virtual crystal approximation [56] to treat the mixing between Co and Fe in atomic sites, which was exploited in a previous study to calculate the *σ*_*xy*_ and *α*_*xy*_ values of Co_3_In_*x*_Sn_2−*x*_S_2_ [57].

## 3. Results and discussion

**Figure 1.**
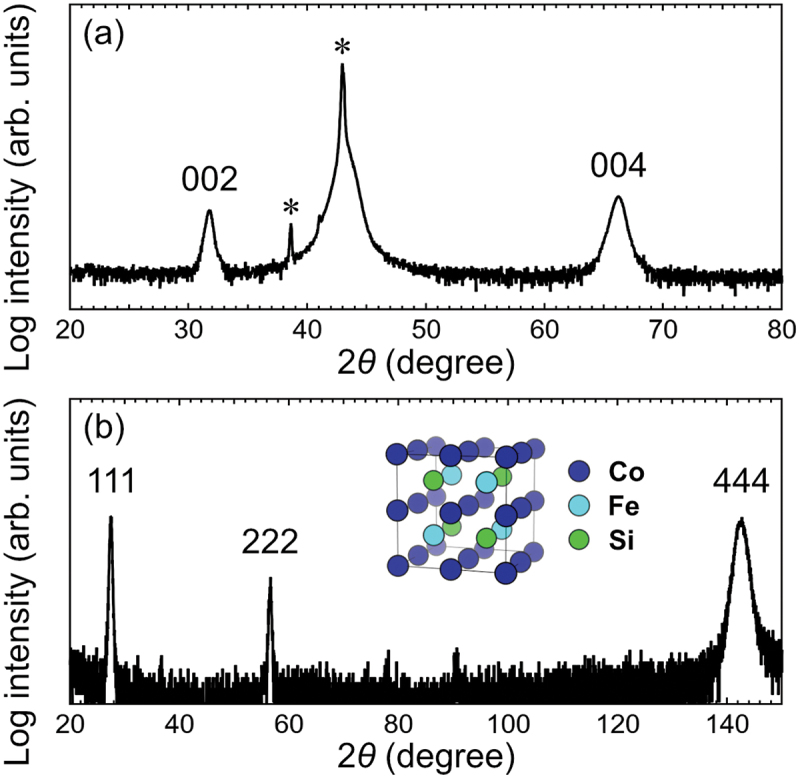
(a) Out-of-plane XRD pattern of the Co_2_FeSi thin film, showing the 002 and 004 diffraction peaks of Co_2_FeSi. The diffraction peaks labeled by * are from the MgO substrate. (b) XRD pattern measured with the film normal tilted out of the X-ray plane by 54.7°. The 111, 222, and 444 diffraction peaks of Co_2_FeSi can be observed. The inset of (b) shows the structure of *L*2_1_-ordered Co_2_FeSi.

The out-of-plane XRD pattern of the Co_2_FeSi thin film is shown in Figure 1(a). Other than the diffraction peaks from the MgO substrate, which are labeled with *, only the 002 and 004 peaks of Co_2_FeSi are visible. The clear 002 superlattice peak suggested the formation of a B2 structure. To confirm the *L*2_1_ structure, we obtained the XRD pattern with the film normal tilted out of the X-ray plane by 54.7°, as shown in Figure 1(b). The clear 111 superlattice peak confirmed the existence of the *L*2_1_ structure, whereas the appearance of diffraction peaks from only the {111} planes indicated the epitaxial growth of Co_2_FeSi. The lattice constant of the Co_2_FeSi thin film was determined to be 5.64 Å based on XRD measurements, which resulted in a lattice mismatch of approximately 5% with the MgO substrate. Such a lattice mismatch typically induces strain at the Co_2_FeSi/MgO interface, leading to defect formation in the interfacial layer of Co_2_FeSi, which would relax the strain. However, because the Co_2_FeSi had a thickness of 41.7 nm, which was much thicker than the strained interfacial layer of Co_2_FeSi, we expected the overall impact of the strain on the thin-film properties to be small. To evaluate the ordering parameters of the B2 and *L*2_1_ structures, the integrated intensity ratios between the 002 and 004 peaks ($I_{002}^{exp}/I_{004}^{exp}$), and between the 111 and 444 peaks ($I_{111}^{exp}/I_{444}^{exp}$), were extracted. These values were compared with the simulated intensity ratios ($I_{002}^{sim}/I_{004}^{sim}$ and $I_{111}^{sim}/I_{444}^{sim}$) of *L*2_1_-ordered Co_2_FeSi, which were calculated using the Visualization for Electronic and STructural Analysis (VESTA) software [58], with the actual composition of the thin film taken into consideration. The degree of B2 order ($S_{B2}=\sqrt{\left(I_{002}^{exp}/I_{004}^{exp}\right)/\left(I_{002}^{sim}/I_{004}^{sim}\right)}$) was estimated to be 1.17, whereas the degree of *L*2_1_ order ($S_{L2_{1}}=\sqrt{\left(I_{111}^{exp}/I_{444}^{exp}\right)/\left(I_{111}^{sim}/I_{444}^{sim}\right)}$) was estimated to be 0.61. These values are comparable to the ordering parameters reported for Co_2_FeSi thin films in previous studies [37,38], indicating that the thin film primarily possessed the *L*2_1_ structure. Meanwhile, some disorder existed in the thin film, which could have influenced the transport properties of Co_2_FeSi. We also measured the in-plane and out-of-plane *M-H* curves of Co_2_FeSi at 10, 100, 200, and 300 K (see the Supplemental Material). The saturation magnetization (*M*_s_) of Co_2_FeSi increased slightly with decreasing *T*. At 10 K, *μ*_0_*M*_s_ = 1.1 T, which corresponded to a magnetic moment of ~ 4.2 *μ*_B_/f.u. This value was smaller than the value of 6 *μ*_B_/f.u. expected for half-metallic Co_2_FeSi at 0 K. As will be shown later, the Co_2_FeSi thin film did not exhibit strong half-metallicity; instead, its *E*_F_ was close to the CB edge. Hence, we expect some deviation in the magnetic moment. In addition, the Co_2_FeSi thin film was deposited directly on an MgO substrate without a buffer layer, and the lattice mismatch between them could lead to the formation of defects and a magnetic dead layer at the Co_2_FeSi/MgO interface. However, the cause of this reduction in magnetic moment is unclear. Notably, the value of *M*_s_ was comparable to some previously reported values obtained using thin films [8,59,60].

**Figure 2.**
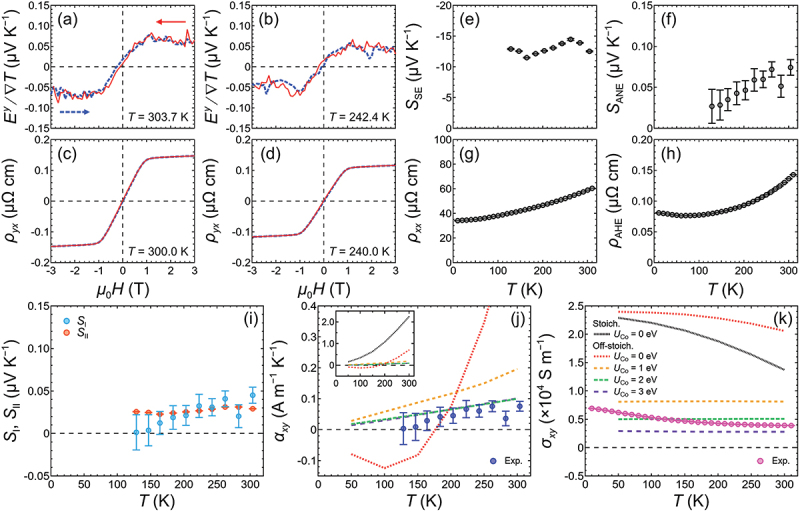
(a) Magnetic field *H* dependence of transverse thermal power (*E*^*y*^/∇*T*) of the Co_2_FeSi thin film measured at *T* = 303.7 and (b) 242.4 K, exhibiting the ANE of Co_2_FeSi. (c) *H* dependence of *ρ*_*yx*_ value of Co_2_FeSi measured at 300.0 K and (d) 240.0 K, exhibiting the AHE of Co_2_FeSi. (e) The measured *S*_SE_, (f) *S*_ANE_, (g) *ρ*_*xx*_, and (h) *ρ*_AHE_ values of Co_2_FeSi as functions of *T*, summarizing the *T* dependences of its transport properties. (i) *T* dependence of the *S*_I_ and *S*_II_ terms, which together constitute the ANE of Co_2_FeSi. (j) *α*_*xy*_ and (k) *σ*_*xy*_ values of Co_2_FeSi as functions of *T* measured in the experiment, in comparison with the calculated results for stoichiometric (Stoich.) and off-stoichiometric (off-stoich.) compositions with various values for the on-site Coulomb interaction at the Co site (*U*_Co_). The results for the off-stoichiometric composition were calculated using a unit cell of (Co_0.95_Fe_0.05_)_2_FeSi, which corresponded to the actual composition of the Co_2_FeSi thin film. The inset of (j) shows the calculated results over a wider range of − 0.5 to + 2.5 A m^−1^ K^−1^ for the *y*-axis. The legends in (k) for the calculated results also apply to (j).

Figure 2(a,b) shows the *H* dependence of *E*^*y*^ divided by ∇*T* measured at *T* = 303.7 and 242.4 K, respectively, exhibiting the ANE of the Co_2_FeSi thin film. These curves are the average of three measurement loops, each obtained with *μ*_0_*H* applied along the out-of-plane direction and swept from + 3 T to − 3 T and then back to + 3 T. Here, *T* is the estimated temperature of Co_2_FeSi corresponding to that of the electrodes used to measure *E*^*y*^, which was calculated using the results of two on-chip thermometers while assuming a linear change in *T* along the thin-film plane. The curves show *H*-odd dependence and saturation at $\left|\mu_{0}H\right|$ ~1 T. Meanwhile, a similar behavior was observed in the *H* dependence of *ρ*_*yx*_ (Figure 2(c,d)), exhibiting the AHE of Co_2_FeSi. It is worth mentioning that the *H* dependence of the ANE and AHE was consistent with the out-of-plane *M-H* curve of the Co_2_FeSi thin film (see the Supplemental Material). We evaluated *S*_ANE_ and *ρ*_AHE_ of Co_2_FeSi by extrapolating the curves at high *H* values after saturation down to zero *H*. The *T* dependences of *S*_ANE_ and *ρ*_AHE_ are shown in Figure 2(f,h), respectively. The value of *S*_ANE_ was positive but small (less than 0.1 μV K^−1^) and decreased with *T* within the range of our thermoelectric measurements (110.9–303.7 K). We were able to separate the different components of ANE and evaluate *α*_*xy*_ using the following expression:

$$S_{\mathrm{ANE}}=S_{I}+S_{\mathrm{II}}=\rho_{\mathrm{xx}}\alpha_{\mathrm{xy}}-S_{\mathrm{SE}}\frac{\rho_{\mathrm{AHE}}}{\rho_{\mathrm{xx}}},$$

along with the measured *S*_SE_ (Figure 2(e)) and longitudinal resistivity (*ρ*_*xx*_) (Figure 2(g)) under zero *H*. Notably, we calculated *ρ*_*xx*_ and *ρ*_AHE_ at the corresponding *T* to match the data for *S*_SE_ and *S*_ANE_ by assuming a linear interpolation between adjacent data points with a spacing of 10 K. As shown in Figure 2(i), the *S*_I_ term, in which *α*_*xy*_ played a crucial role, followed the trend of the *T* dependence of *S*_ANE_ and decreased as *T* decreased towards zero. By contrast, the *S*_II_ term, which was due to the AHE acting on the longitudinal charge current induced by the SE, showed minimal *T* dependence. Overall, the *S*_I_ and *S*_II_ terms were comparable in magnitude. Figure 2(j) shows *α*_*xy*_ as a function of *T*. The value of *α*_*xy*_ was less than 0.1 A m^−1^ K^−1^ at room temperature, and decreased slightly with *T*. Comparing to the predicted value of 2.57 A m^−1^ K^−1^ for *α*_*xy*_ at 300 K without considering *U* [48], there was a clear discrepancy, as the values obtained in the experiment were more than one order of magnitude smaller. We also obtained the *σ*_*xy*_ values of Co_2_FeSi using the equation $\sigma_{\mathrm{xy}}=\rho_{\mathrm{AHE}}/\left(\rho_{xx}^{2}+\rho_{AHE}^{2}\right)$, as shown in Figure 2(k). The obtained values of *ρ*_*xx*_ and *ρ*_AHE_ were consistent with those found in previous studies [61,62].

**Figure 3.**
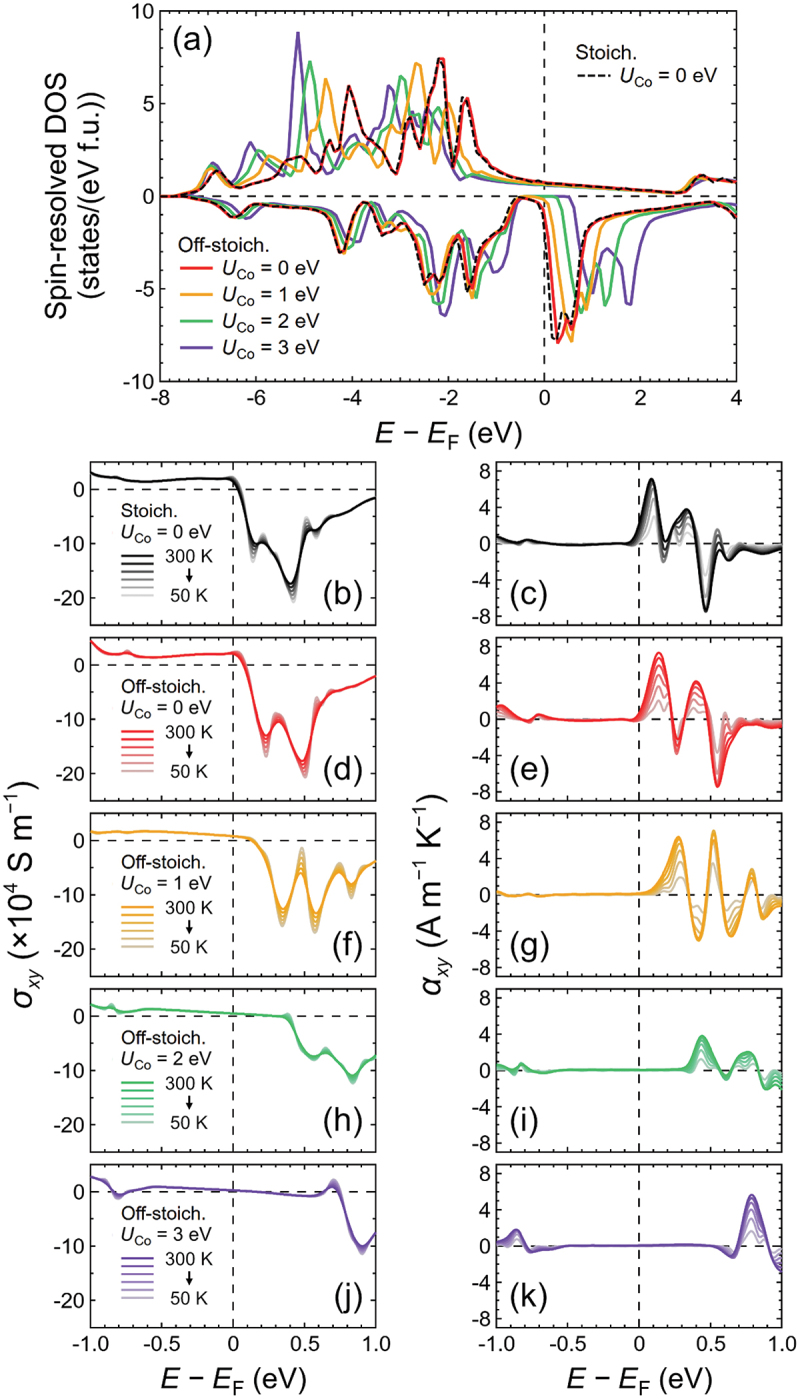
(a) Spin-resolved DOS values for Co_2_FeSi with stoichiometric (Stoich.) and off-stoichiometric (off-stoich.) compositions around the Fermi level (*E*_F_), with various values for the on-site Coulomb interaction at the Co site (*U*_Co_). The results for the off-stoichiometric composition were calculated using a unit cell of (Co_0.95_Fe_0.05_)_2_FeSi, which corresponded to the actual composition of the Co_2_FeSi thin film. (b) Calculated *σ*_*xy*_ values of the stoichiometric Co_2_FeSi in the energy window of ±1 eV relative to *E*_F_ with *U*_Co_ = 0 eV, and values of off-stoichiometric Co_2_FeSi with (d) *U*_Co_ = 0 eV, (f) *U*_Co_ = 1 eV, (h) *U*_Co_ = 2 eV, and (j) *U*_Co_ = 3 eV. (c) Corresponding *α*_*xy*_ values of stoichiometric Co_2_FeSi with *U*_Co_ = 0 eV, and off-stoichiometric Co_2_FeSi with (e) *U*_Co_ = 0 eV, (g) *U*_Co_ = 1 eV, (i) *U*_Co_ = 2 eV, and (k) *U*_Co_ = 3 eV. The saturation of colors of the lines indicates the *T* values of the results, which ranged from 50 to 300 K in 50 K steps. The values of *α*_*xy*_ and *σ*_*xy*_ at *E*_F_ in (b)–(k) as functions of *T* are shown in Figure 2(j, k), respectively.

To gain insight into this discrepancy, we evaluated the *σ*_*xy*_ and *α*_*xy*_ values of Co_2_FeSi using first-principles calculations. Because the Co_2_FeSi thin film used in the experiment contained approximately 10% more Fe than the stoichiometric composition, we first studied the effect of this extra Fe using a unit cell of (Co_0.95_Fe_0.05_)_2_FeSi. Here, the calculated results for (Co_0.95_Fe_0.05_)_2_FeSi are referred to as off-stoichiometric in this study. The spin-resolved density of states (DOS) values of the stoichiometric and off-stoichiometric compositions with *U*_Co_ = 0 eV are shown in Figure 3(a) for comparison. The CB edge of the minority spin shifted slightly to higher energy for the off-stoichiometric composition. Figure 3(b) (Figure 3(d)) shows the *σ*_*xy*_ values of the stoichiometric (off-stoichiometric) composition around *E*_F_ at *T* values ranging from 50 to 300 K in 50 K steps, while the corresponding *α*_*xy*_ values are shown in Figure 3(c) (Figure 3(e)). *E*_F_ crossed the lower energy side of a peak in *α*_*xy*_, resulting in large values and substantial *T* dependences for both cases, as shown in Figure 3(c, e). The *T* dependences of *α*_*xy*_ and *σ*_*xy*_ at *E*_F_ are plotted in Figure 2(j, k), respectively, in comparison to the experimental results. For the stoichiometric composition, *α*_*xy*_ = 2.3 A m^−1^ K^−1^ at 300 K (inset of Figure 2(j)), which was similar to the value predicted in a previous study [48]. After taking the composition into consideration, *α*_*xy*_ exhibited a clear decrease to 0.7 A m^−1^ K^−1^ at 300 K for the off-stoichiometric composition, although it was still one order of magnitude larger than the *α*_*xy*_ value obtained in the experiment. We then considered the effect of *U*_Co_, and performed first-principles calculations for Co_2_FeSi with *U*_Co_ = 1, 2, and 3 eV. The spin-resolved DOS values of the off-stoichiometric composition with various *U*_Co_ values are summarized in Figure 3(a) as well. The CB edge of the minority spin exhibited a substantial shift toward higher energy with increasing *U*_Co_. *E*_F_ lay in the middle of the bandgap in the minority-spin channel when *U*_Co_ = 3 eV, exhibiting strong half-metallicity, which agreed with previously calculated results [26]. Figure 3(f–k) shows *σ*_*xy*_ and *α*_*xy*_ values around *E*_F_ at different *T* values for the off-stoichiometric composition with finite *U*_Co_. Compared to the case with zero *U*_Co_, the peak responsible for large *α*_*xy*_ also shifted to higher energy with increasing *U*_Co_. Consequently, the *α*_*xy*_ values at *E*_F_ further decreased to close to zero and exhibited minimal *T* dependence, as shown in Figure 2(j). The calculated *α*_*xy*_ values agreed well with the experimental results especially when *U*_Co_ > 1 eV. Meanwhile, the values of *σ*_*xy*_ obtained in the experiment were closer to the calculated results when a finite *U*_Co_ was considered, as shown in Figure 2(k). However, the trend of a slight decrease in *σ*_*xy*_ with increasing *T* observed in the experiment was not reproduced in the calculation. Notably, the calculated *α*_*xy*_ only accounted for the intrinsic contribution originating from the Berry curvature. Extrinsic mechanisms, such as skew scattering and side-jump, may have contributed to the *α*_*xy*_ value of the Co_2_FeSi thin film in the experiment, although they were not theoretically considered in this study. The temperature dependence of *α*_*xy*_ obtained in the experiment agreed well with the calculated results, suggesting that the ANE of the Co_2_FeSi thin film can be explained by the intrinsic contribution of *α*_*xy*_ originated from the Berry curvature. A mostly identical behavior in spin-resolved DOS, *σ*_*xy*_, and *α*_*xy*_ values were observed for the stoichiometric composition with finite *U*_Co_ (see the Supplemental Material). These results suggest that *U*_Co_ should be taken into consideration when describing the *σ*_*xy*_ and *α*_*xy*_ values of Co_2_FeSi.

**Figure 4.**
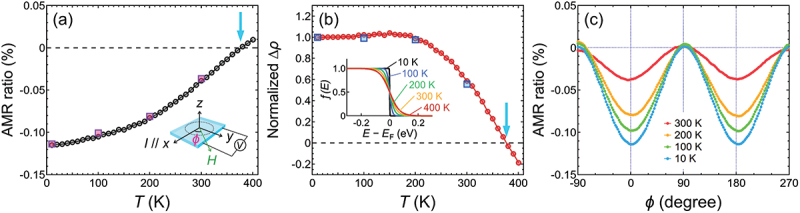
(a) AMR ratio $\left(=(\rho_{∥}-\rho_{⊥})/\rho_{⊥}\times 100\%\right)$ of the Co_2_FeSi thin film measured from 10 to 400 K. The inset shows a schematic of the AMR measurement showing the definition of Φ to represent the direction of *H*. (b) Δρ ($\rho_{∥}-\rho_{⊥}$) values of Co_2_FeSi normalized by the value at 10 K as a function of *T*. The inset illustrates the Fermi distribution function (*f*(*E*)) at different *T* values. The cyan arrows in (a) and (b) mark the sign changes in the AMR ratio and normalized Δρ at *T* ~377 K. (c) AMR ratio as a function of Φ measured at various *T*. The values obtained from (c) are plotted in (a) and (b) as squares at the corresponding *T* for comparison.

As *U*_Co_ increased, the spin-resolved DOS of Co_2_FeSi showed an increase in energy for the CB edge, exhibiting a clear bandgap in the minority-spin channel at *E*_F_ (Figure 3(a)). However, this was not supported by experimental observations. We measured the AMR effect using the same sample to study the electronic band structure around *E*_F_ of Co_2_FeSi. We applied an electrical current *I* = 1 mA along the [100] direction of the MgO substrate (|| [110] direction of Co_2_FeSi) and measured the longitudinal resistivity using four-terminal sensing. A *μ*_0_*H* value of 0.3 T was applied within the thin film plane during the measurement, and Φ was used to define the direction of *H*, where Φ = 0 indicated that *H* || *I* (inset of Figure 4(a)). In addition to the conventional method of determining the AMR ratio by sweeping Φ from 0° to 360° (Figure 4(c)), we also used a different method, *i.e*., only measuring the longitudinal resistivity at Φ = 0° ($\rho_{∥}$) and Φ = 90° ($\rho_{⊥}$) at different *T* values, to obtain the *T* dependence of AMR with better detail. The AMR ratio $\left(=(\rho_{∥}-\rho_{⊥})/\rho_{⊥}\times 100\%\right)$ as a function of *T* from 10 to 400 K is shown in Figure 4(a). The results obtained using the conventional method are shown in Figure 4(a) for comparison, where a quantitative agreement can be seen, demonstrating the validity of this method. The AMR ratio was negative at low *T*, increased monotonically with increasing *T*, and eventually changed sign to positive at ~ 377 K. To further gain insight into the electronic structure of the Co_2_FeSi thin film, we calculated the *T* dependence of $\Delta \rho =\rho_{∥}-\rho_{⊥}$ normalized by the value at 10 K, as shown in Figure 4(b). As seen, Δρ showed minimal *T* dependence from 10 to ~ 200 K, after which it exhibited a clear trend of decreasing in magnitude with increasing *T*. Following the modeling of the AMR effect proposed by Kokado et al. [29] and its application to the AMR effect of the half-metallic Heusler alloy Co_2_FeGa_0.5_Ge_0.5_ [31], Δρ can be approximately proportional to $\rho_{\text{s}{,}_{↑}\;\to \text{ d}{,}_{↓}}-\;\rho \text{ s}{,}_{↑}\;\to \text{ d}{,}_{↑}$, where $\rho_{s{,}_{↑}\text{\textbackslash{}tod }\to \;\text{\textbackslash{}tod}d{,}_{↓}}$ ($\rho_{s{,}_{↑}\text{\textbackslash{}tod }\to \;\text{\textbackslash{}tod}d{,}_{↑}}$) is the resistivity for the *s-d* scattering from the *s* electron of ↑ spin to the localized *d* states of ↓ spin (↑ spin). If we ignore the parameters that are negligibly affected by *T*, $\rho_{s{,}_{↑}\text{\textbackslash{}tod }\to \;\text{\textbackslash{}tod}d{,}_{↓}}$ ($\rho_{s{,}_{↑}\text{\textbackslash{}tod }\to \;\text{\textbackslash{}tod}d{,}_{↑}}$) can be further simplified to be proportional to $D_{↓}^{\left(d\right)}\left(E_{F}\right)$ ($D_{↑}^{\left(d\right)}\left(E_{F}\right)$). Here, $D_{↓}^{\left(d\right)}\left(E_{F}\right)$ ($D_{↑}^{\left(d\right)}\left(E_{F}\right)$) represents the DOS of the *d* states of ↓ spin (↑ spin) around *E*_F_ that contributes to the *s-d* scattering, while also having the thermally excited electron occupation following the Fermi distribution function (*f*(*E*)) taken into consideration (inset of Figure 4(b)). As seen in the case of Co_2_FeGa_0.5_Ge_0.5_, the DOS of the *d* states of ↑ spin is nearly independent of energy around *E*_F_, and the *T* dependence of Δρ is mainly due to $D_{↓}^{\left(d\right)}\left(E_{F}\right)$. As a result, Δρ being independent of *T* suggests that *E*_F_ is located around the center of the bandgap in the minority-spin channel; whereas clear decrease in the normalized Δρ with increasing *T* would indicate that *E*_F_ lies close to a band edge that is adjacent to the bandgap, or there are in-gap states formed in the bandgap [31]. In line with this argument, Figure 4(b) suggests that the *E*_F_ of Co_2_FeSi lies in the bandgap in the minority-spin channel but is relatively close to the CB edge. For comparison, the CB edge of the minority spin of *L*2_1_-ordered Co_2_FeGa_0.5_Ge_0.5_ was estimated to be ~0.3 eV above *E*_F_ using first-principles calculations, while its normalized Δρ showed no decrease with increasing *T* up to 300 K [31]. Therefore, the observed *T* dependence of the normalized Δρ of the Co_2_FeSi thin film would contradict the CB edge of the minority spin being further away from *E*_F_ than Co_2_FeGa_0.5_Ge_0.5_. The calculated spin-resolved DOS with *U*_Co_ values of 2 and 3 eV showed that the CB edge was at approximately 0.3 and 0.6 eV above *E*_F_, respectively (Figure 3(a)). Thus, the *T* dependence of the normalized Δρ of the AMR effect sets an upper boundary for the *U*_Co_ value of the Co_2_FeSi thin film to be smaller than 2 eV. Meanwhile, in comparison with the calculated results, the experimentally measured *T* dependence of *α*_*xy*_ sets a lower boundary for the *U*_Co_ value to be larger than 1 eV. Overall, our results suggested that a nonzero but small *U*_Co_ of approximately 1–2 eV is appropriate for describing the transport properties of Co_2_FeSi.

## 4. Conclusions

We systematically studied the transport properties of Co_2_FeSi thin films at various *T* values. The experimentally measured *α*_*xy*_ values were positive but less than 0.1 A m^−1^ K^−1^, and only exhibits a slight decrease with decreasing *T*. This led to a small *S*_ANE_ of less than 0.1 μV K^−1^. This behavior agreed with the theoretical prediction if a *U*_Co_ value larger than 1 eV was considered in the first-principles calculations. On the other hand, the *T* dependence of Δρ due to the AMR effect suggested that *E*_F_ of Co_2_FeSi fell in the bandgap in the minority-spin channel but was close to the CB edge. This contradicted the theoretical prediction when a *U*_Co_ value of 2 eV or higher was incorporated in the calculations. Therefore, a *U*_Co_ value of approximately 1–2 eV was found to be appropriate to explain the experimental results. Our findings demonstrated that measuring the *T* dependence of the anomalous Nernst conductivity and anisotropic magnetoresistance could be a powerful tool for studying the electronic band structure close to *E*_F_ and probing the effect of on-site Coulomb interaction. In addition, our results suggest that the effect of on-site Coulomb interaction should be examined when calculating the electronic band structure and transport properties of Co-based Heusler alloys.

## Supplementary Material

Supplemental Material

## References

[1] Brown PJ, Neumann KU, Webster PJ, et al. The magnetization distributions in some Heusler alloys proposed as half-metallic ferromagnets. J Phys Condens Matter. 2000;12:1827. doi: 10.1088/0953-8984/12/8/325

[2] Picozzi S, Continenza A, Freeman AJ. Co_2_Mn*X* (*X* = Si, Ge, Sn) Heusler compounds: an *Ab initio* study of their structural, electronic, and magnetic properties at zero and elevated pressure. Phys Rev B. 2002;66(9):094421. doi: 10.1103/PhysRevB.66.094421

[3] Galanakis I, Dederichs PH, Papanikolaou N. Slater-Pauling behavior and origin of the half-metallicity of the full-Heusler alloys. Phys Rev B. 2002;66(17):174429. doi: 10.1103/PhysRevB.66.174429

[4] Kübler J, Fecher GH, Felser C. Understanding the trend in the Curie temperatures of Co_2_-based Heusler compounds: *Ab initio* calculations. Phys Rev B. 2007;76(2):024414. doi: 10.1103/PhysRevB.76.024414

[5] Kandpal HC, Fecher GH, Felser C. Calculated electronic and magnetic properties of the half-metallic, transition metal based Heusler compounds. J Phys D Appl Phys. 2007;40(6):1507. doi: 10.1088/0022-3727/40/6/S01

[6] Sakuraba Y, Hattori M, Oogane M, et al. Giant tunneling magnetoresistance in Co_2_MnSi/Al–O/Co_2_MnSi magnetic tunnel junctions. Appl Phys Lett. 2006;88(19):192508. doi: 10.1063/1.2202724

[7] Li S, Takahashi YK, Furubayashi T, et al. Enhancement of giant magnetoresistance by L2_1_ ordering in Co_2_Fe(Ge_0.5_Ga_0.5_) Heusler alloy current-perpendicular-to-plane pseudo spin valves. Appl Phys Lett. 2013;103(4):042405. doi: 10.1063/1.4816382

[8] Kimura T, Hashimoto N, Yamada S, et al. Room-temperature generation of giant pure spin currents using epitaxial Co_2_FeSi spin injectors. NPG Asia Mater. 2012;4(3):e9. doi: 10.1038/am.2012.16

[9] Kasahara K, Fujita Y, Yamada S, et al. Greatly enhanced generation efficiency of pure spin currents in Ge using Heusler compound Co_2_FeSi electrodes. Appl Phys Express. 2014;7(3):033002. doi: 10.7567/APEX.7.033002

[10] Inomata K, Ikeda N, Tezuka N, et al. Highly spin-polarized materials and devices for spintronics. Sci Technol Adv Mater. 2008;9(1):014101. doi: 10.1088/1468-6996/9/1/014101 27877927 PMC5099796

[11] Felser C, Wollmann L, Chadov S, et al. Basics and prospective of magnetic Heusler compounds. APL Mater. 2015;3(4):041518. doi: 10.1063/1.4917387

[12] Elphick K, Frost W, Samiepour M, et al. Heusler alloys for spintronic devices: review on recent development and future perspectives. Sci Technol Adv Mater. 2021;22(1):235–11. doi: 10.1080/14686996.2020.1812364 33828415 PMC8009123

[13] Kübler J, Felser C. Weyl points in the ferromagnetic Heusler compound Co_2_MnAl. Europhys Lett. 2016;114(4):47005. doi: 10.1209/0295-5075/114/47005

[14] Chang G, Xu SY, Zhou X, et al. Topological Hopf and chain link semimetal states and their application to Co_2_MnGa. Phys Rev Lett. 2017;119(15):156401. doi: 10.1103/PhysRevLett.119.156401 29077460

[15] Noky J, Gooth J, Felser C, et al. Characterization of topological band structures away from the Fermi level by the anomalous Nernst effect. Phys Rev B. 2018;98(24):241106(R). doi: 10.1103/PhysRevB.98.241106

[16] Belopolski I, Manna K, Sanchez DS, et al. Discovery of topological Weyl fermion lines and drumhead surface states in a room temperature magnet. Science. 2019;365(6459):1278–1281. doi: 10.1126/science.aav2327 31604235

[17] Sumida K, Sakuraba Y, Masuda K, et al. Spin-polarized Weyl cones and giant anomalous Nernst effect in ferromagnetic Heusler films. Commun Mater. 2020;1(1):89. doi: 10.1038/s43246-020-00088-w

[18] Li P, Koo J, Ning W, et al. Giant room temperature anomalous Hall effect and tunable topology in a ferromagnetic topological semimetal Co_2_MnAl. Nat Commun. 2020;11(1):3476. doi: 10.1038/s41467-020-17174-9 32651362 PMC7351740

[19] Sakai A, Mizuta YP, Nugroho AA, et al. Giant anomalous Nernst effect and quantum-critical scaling in a ferromagnetic semimetal. Nat Phys. 2018;14(11):1119. doi: 10.1038/s41567-018-0225-6

[20] Guin SN, Manna K, Noky J, et al. Anomalous Nernst effect beyond the magnetization scaling relation in the ferromagnetic Heusler compound Co_2_MnGa. NPG Asia Mater. 2019;11(1):16. doi: 10.1038/s41427-019-0116-z

[21] Xu L, Li X, Ding L, et al. Anomalous transverse response of Co _2_ MnGa and universality of the room-temperature $\alpha_{i}^{A}j/\alpha_{i}^{A}j$ ratio across topological magnets. Phys Rev B. 2020;101(18):180404. doi: 10.1103/PhysRevB.101.180404

[22] Hu J, Butler T, Cabero ZMA, et al. Regulating the anomalous Hall and Nernst effects in Heusler-based trilayers. Appl Phys Lett. 2020;117(6):062405. doi: 10.1063/5.0014879

[23] Uchida K, Zhou W, Sakuraba Y. Transverse thermoelectric generation using magnetic materials. Appl Phys Lett. 2021;118(14):140504. doi: 10.1063/5.0046877

[24] Nakatani T, Kulkarni PD, Suto H, et al. Perspective on nanoscale magnetic sensors using giant anomalous Hall effect in topological magnetic materials for read head application in magnetic recording. Appl Phys Lett. 2024;124(7):070501. doi: 10.1063/5.0191974

[25] Wurmehl S, Fecher CH, Kandpal HC, et al. Geometric, electronic, and magnetic structure of Co_2_FeSi: Curie temperature and magnetic moment measurements and calculations. Phys Rev B. 2005;72(18):184434. doi: 10.1103/PhysRevB.72.184434

[26] Kandpal HC, Fecher GH, Felser C, et al. Correlation in the transition-metal-based Heusler compounds Co_2_MnSi and Co_2_FeSi. Phys Rev B. 2006;73(9):094422. doi: 10.1103/PhysRevB.73.094422

[27] Gercsi Z, Hono K. Ab initio predictions for the effect of disorder and quarternary alloying on the half-metallic properties of selected Co_2_Fe-based Heusler alloys. J Phys Condens Matter. 2007;19(32):326216. doi: 10.1088/0953-8984/19/32/326216

[28] Bombor D, Blum CGF, Volkonskiy O, et al. Half-metallic ferromagnetism with unexpectedly small spin splitting in the Heusler compound Co_2_FeSi. Phys Rev Lett. 2013;110(6):066601. doi: 10.1103/PhysRevLett.110.066601 23432284

[29] Kokado S, Tsunoda M, Harigaya K, et al. Anisotropic magnetoresistance effects in Fe, Co, Ni, Fe_4_N, and half-metallic ferromagnet: a systematic analysis. J Phys Soc Jpn. 2012;81(2):024705. doi: 10.1143/JPSJ.81.024705

[30] Sakuraba Y, Kokado S, Hirayama Y, et al. Quantitative analysis of anisotropic magnetoresistance in Co_2_MnZ and Co_2_FeZ epitaxial thin films: a facile way to investigate spin-polarization in half-metallic Heusler compounds. Appl Phys Lett. 2014;104(17):172407. doi: 10.1063/1.4874851

[31] Kushwaha VK, Kokado S, Kasai S, et al. Prediction of half-metallic gap formation and Fermi level position in Co-based Heusler alloy epitaxial thin films through anisotropic magnetoresistance effect. Phys Rev Mater. 2022;6(6):064411. doi: 10.1103/PhysRevMaterials.6.064411

[32] Gercsi Z, Rajanikanth A, Takahashi YK, et al. Spin polarization of Co_2_FeSi full-Heusler alloy and tunneling magnetoresistance of its magnetic tunneling junctions. Appl Phys Lett. 2006;89(8):082512. doi: 10.1063/1.2338025

[33] Karthik SV, Rajanikanth A, Nakatani TM, et al. Effect of Cr substitution for Fe on the spin polarization of Co2Cr*_x_*Fe_1−*x*_Si Heusler alloys. J Appl Phys. 2007;102(4):043903. doi: 10.1063/1.2769175

[34] Makinistian L, Faiz MM, Panguluri RP, et al. On the half-metallicity of Co_2_FeSi Heusler alloy: point-contact Andreev reflection spectroscopy and *ab initio* study. Phys Rev B. 2013;87(22):220402. doi: 10.1103/PhysRevB.87.220402

[35] Huang HL, Tung JC, Guo GY. Anomalous Hall effect and current spin polarization in Co_2_Fe*X* Heusler compounds (*X* = Al, Ga, In, Si, Ge, and Sn): a systematic *Ab initio* study. Phys Rev B. 2015;91(13):134409. doi: 10.1103/PhysRevB.91.134409

[36] Oogane M, Shinano M, Sakuraba Y, et al. Tunnel magnetoresistance effect in magnetic tunnel junctions using epitaxial Co_2_FeSi Heusler alloy electrode. J Appl Phys. 2009;105(7):07C903. doi: 10.1063/1.3062814

[37] Chen J, Sakuraba Y, Masuda K, et al. Enhancement of L2_1_ order and spin-polarization in Co_2_FeSi thin film by substitution of Fe with Ti. Appl Phys Lett. 2017;110(24):242401. doi: 10.1063/1.4985237

[38] Sumida K, Fujita Y, Zhou W, et al. Role of on-site Coulomb interactions in the half-metallic Weyl ferromagnet candidate thin-film Co_2_FeSi. Phys Rev B. 2023;108(24):L241101. doi: 10.1103/PhysRevB.108.L241101

[39] Xiao D, Yao Y, Fang Z, et al. Berry-phase effect in anomalous thermoelectric transport. Phys Rev Lett. 2006;97(2):026603. doi: 10.1103/PhysRevLett.97.026603 16907470

[40] Guin SN, Vir P, Zhang Y, et al. Zero-field Nernst effect in a ferromagnetic kagome-lattice Weyl-semimetal Co_3_Sn_2_S_2_. Adv Mater. 2019;31(25):1806622. doi: 10.1002/adma.201806622 31044469

[41] Ding L, Koo J, Xu L, et al. Intrinsic anomalous Nernst effect amplified by disorder in a half-metallic semimetal. Phys Rev X. 2019;9:041061. doi: 10.1103/PhysRevX.9.041061

[42] Yang H, You W, Wang J, et al. Giant anomalous Nernst effect in the magnetic Weyl semimetal Co_3_Sn_2_S_2_. Phys Rev Mater. 2020;4(2):024202. doi: 10.1103/PhysRevMaterials.4.024202

[43] Wuttke C, Caglieris F, Sykora S, et al. Berry curvature unravelled by the anomalous Nernst effect in Mn_3_Ge. Phys Rev B. 2019;100(8):085111. doi: 10.1103/PhysRevB.100.085111

[44] Sakuraba Y, Hyodo K, Sakuma A, et al. Giant anomalous Nernst effect in the Co_2_MnAl_1−*x*_Si*_x_* Heusler alloy induced by Fermi level tuning and atomic ordering. Phys Rev B. 2020;101(13):134407. doi: 10.1103/PhysRevB.101.134407

[45] Sakai A, Minami S, Koretsune T, et al. Iron-based binary ferromagnets for transverse thermoelectric conversion. Nature. 2020;581(7806):53–57. doi: 10.1038/s41586-020-2230-z 32376952

[46] Asaba T, Ivanov V, Thomas SM, et al. Colossal anomalous Nernst effect in a correlated noncentrosymmetric kagome ferromagnet. Sci Adv. 2021;7(13):eabf1467. doi: 10.1126/sciadv.abf1467 33771869 PMC7997519

[47] Breidenbach AT, Yu H, Peterson TA, et al. Anomalous Nernst and Seebeck coefficients in epitaxial thin film Co_2_MnAl*_x_*Si_1−*x*_ and Co_2_FeAl. Phys Rev B. 2022;105(14):144405. doi: 10.1103/PhysRevB.105.144405

[48] Noky J, Zhang Y, Gooth J, et al. Giant anomalous Hall and Nernst effect in magnetic cubic Heusler compounds. npj Comput Mater. 2020;6(1):77. doi: 10.1038/s41524-020-0342-5

[49] Zhou W, Miura A, Sakuraba Y, et al. Direct electrical probing of anomalous Nernst conductivity. Phys Rev Appl. 2023;19(6):064079. doi: 10.1103/PhysRevApplied.19.064079

[50] Kresse G, Furthmüller J. Efficient iterative schemes for *Ab initio* total-energy calculations using a plane-wave basis set. Phys Rev B. 1996;54(16):11169–11186. doi: 10.1103/PhysRevB.54.11169 9984901

[51] Perdew JP, Burke K, Ernzerhof M. Generalized gradient approximation made simple. Phys Rev Lett. 1996;77(18):3865–3868. doi: 10.1103/PhysRevLett.77.3865 10062328

[52] Blöchl PE. Projector augmented-wave method. Phys Rev B. 1994;50(24):17953–17979. doi: 10.1103/PhysRevB.50.17953 9976227

[53] Kresse G, Joubert D. From ultrasoft pseudopotentials to the projector augmented-wave method. Phys Rev B. 1999;59(3):1758–1775. doi: 10.1103/PhysRevB.59.1758

[54] Yao Y, Kleinman L, MacDonald AH, et al. First principles calculation of anomalous Hall conductivity in ferromagnetic bcc Fe. Phys Rev Lett. 2004;92(3):037204. doi: 10.1103/PhysRevLett.92.037204 14753904

[55] Goto K, Kumara LSR, Sakuraba Y, et al. Effects of the atomic order on the half-metallic electronic structure in the Co_2_Fe(Ga_0.5_Ge_0.5_) Heusler alloy thin film. Phys Rev Mater. 2020;4(11):114406. doi: 10.1103/PhysRevMaterials.4.114406

[56] Bellaiche L, Vanderbilt D. Virtual crystal approximation revisited: application to dielectric and piezoelectric properties of perovskites. Phys Rev B. 2000;61(12):7877–7882. doi: 10.1103/PhysRevB.61.7877

[57] Yanagi Y, Ikeda J, Fujiwara K, et al. First-principles investigation of magnetic and transport properties in hole-doped shandite compounds Co_3_In*_x_*Sn_2−*x*_S_2_. Phys Rev B. 2021;103(20):205112. doi: 10.1103/PhysRevB.103.205112

[58] Momma K, Izumi F. *Vesta 3* for three-dimensional visualization of crystal, volumetric and morphology data. J Appl Crystallogr. 2011;44(6):1272–1276. doi: 10.1107/S0021889811038970

[59] Oogane M, Yilgin R, Shinano M, et al. Magnetic damping constant of Co_2_FeSi Heusler alloy thin film. J Appl Phys. 2007;101(9):09J501. doi: 10.1063/1.2709751

[60] Schneider H, Herbort C, Jakob G, et al. Structural, magnetic and transport properties of Co_2_FeSi Heusler films. J Phys D Appl Phys. 2007;40(6):1548. doi: 10.1088/0022-3727/40/6/S06

[61] Imort IM, Thomas P, Reiss G, et al. Anomalous Hall effect in the Co-based Heusler compounds Co_2_FeSi and Co_2_FeAl. J Appl Phys. 2012;111(7):07D313. doi: 10.1063/1.3678323

[62] Yadav A, Chaudhary S. Effect of growth temperature on the electronic transport and anomalous Hall effect response in co-sputtered Co_2_FeSi thin films. J Appl Phys. 2015;118(19):193902. doi: 10.1063/1.4935823

