# Supplementary material for: Comparative experimental and theoretical study on anomalous Nernst effect of Heusler alloy Co2FeSi thin film: estimation of on-site Coulomb interaction at Co site
Source: Sci Technol Adv Mater. 2025 Sep 22;26(1):2564061. doi: 10.1080/14686996.2025.2564061 (PMC12570244; doi:10.1080/14686996.2025.2564061)
Supplement: Supplemental Material [file TSTA_A_2564061_SM7324.docx]

Supplemental Material

Comparative experimental and theoretical study on anomalous Nernst effect of Heusler alloy Co_2_FeSi thin film: Estimation of on-site Coulomb interaction at Co

# site

Weinan Zhou^a^, Keisuke Masuda^a^, Kazuki Sumida^b^, Yuichi Fujita^a*^, Akio Kimura^c,d,e^, and Yuya Sakuraba^a^

^a^Research Center for Magnetic and Spintronic Materials (CMSM), National Institute for Materials Science (NIMS), Tsukuba, Ibaraki, Japan;

^b^Research Institute for Synchrotron Radiation Science, Hiroshima University, Higashi-Hiroshima, Hiroshima, Japan;

^c^Graduate School of Advanced Science and Engineering, Hiroshima University, HigashiHiroshima, Hiroshima, Japan;

^d^International Institute for Sustainability with Knotted Chiral Meta Matter (WPI-SKCM^2^), Higashi-Hiroshima, Hiroshima, Japan;

^e^Synchrotron Radiation Research Center, National Institutes for Quantum Science and Technology (QST), Sayo, Hyogo, Japan;

Contact:

Weinan Zhou ZHOU.Weinan@nims.go.jp; Yuya Sakuraba SAKURABA.Yuya@nims.go.jp

Research Center for Magnetic and Spintronic Materials (CMSM), National Institute for Materials Science (NIMS), Tsukuba, Ibaraki 305-0047, Japan

*Present address: Global Research and Development Center for Business by Quantum-AI technology (G-QuAT), National Institute of Advanced Industrial Science and Technology (AIST),

Tsukuba, Ibaraki 305-8568, Japan


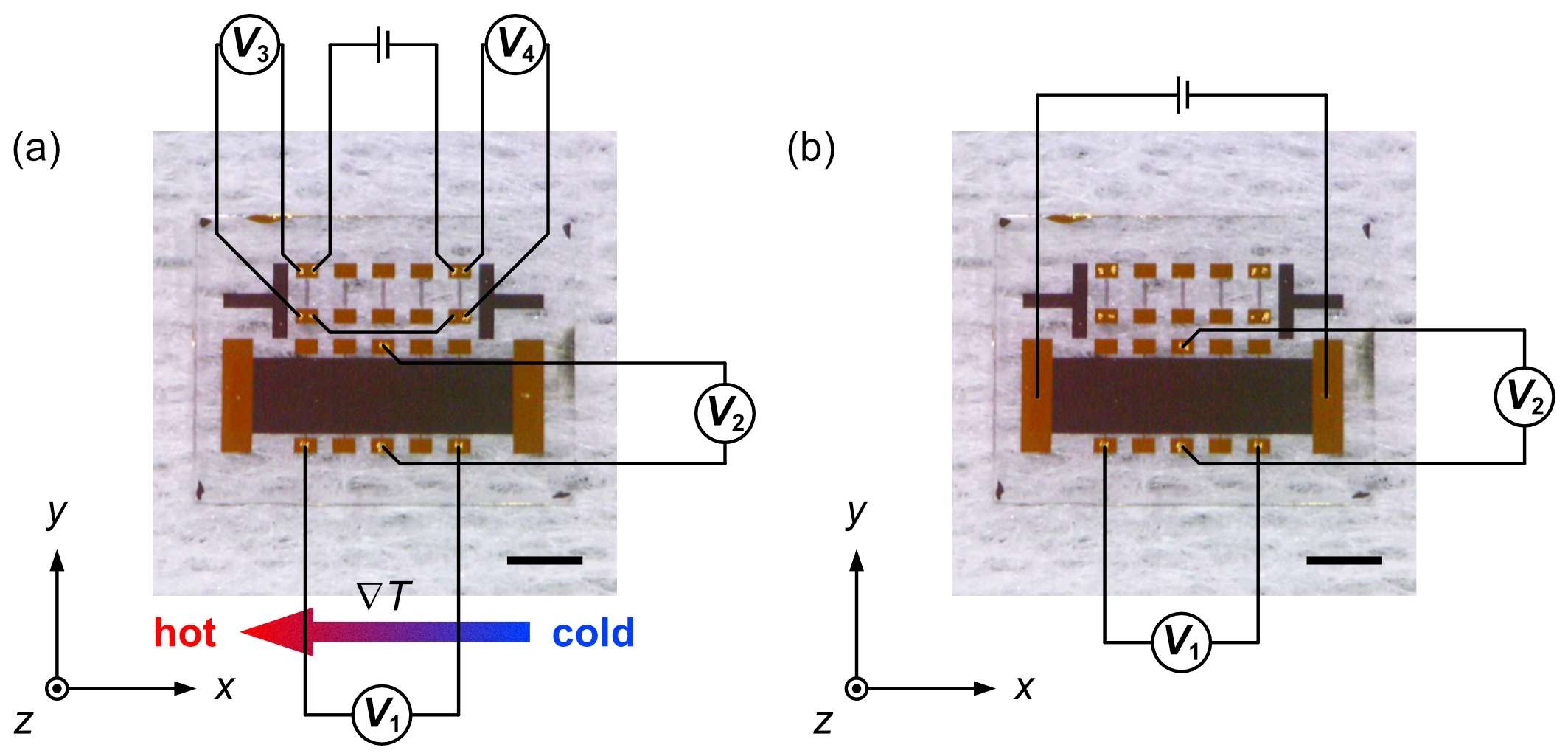


Figure S1. (a) Photograph of the patterned sample, together with the schematic representation of the experimental configuration for the ANE and (b) AHE measurements. The corresponding Cartesian coordinate system is also shown. In (a), V_1_, V_2_, V_3_, and V_4_ represent four nanovoltmeters measuring the longitudinal thermoelectric signal due to SE, the transverse thermoelectric signal due to ANE, the resistance of on-chip thermometers at the hot side and cold side of the sample, respectively. In (b), V_1_ and V_2_ represent 2 nanovoltmeters for measuring the longitudinal and transverse resistance, respectively. The scale bars in (a) and (b) indicate the length of 2 mm.


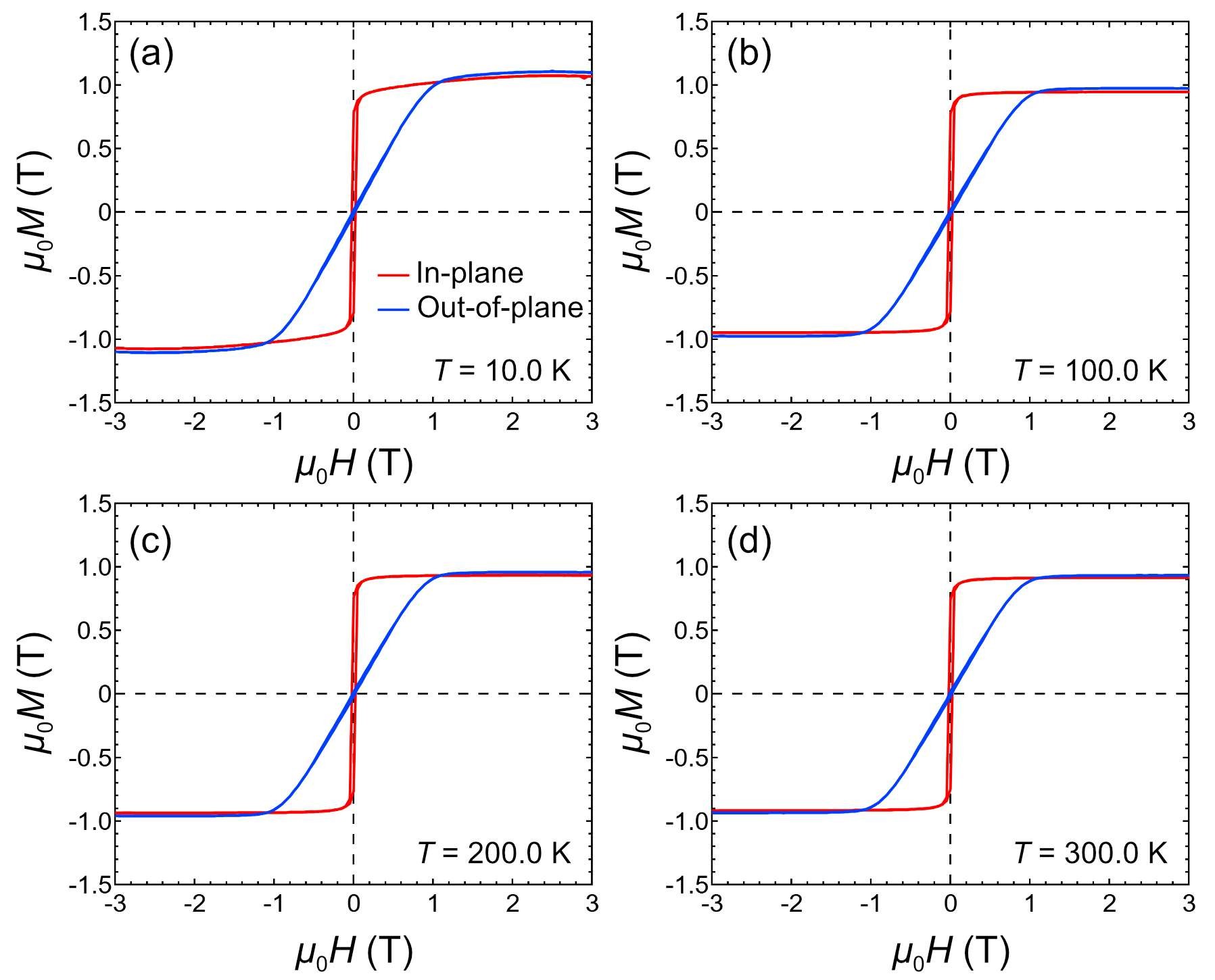


Figure R2. (a) In-plane and out-of-plane M-H curves of the Co_2_FeSi thin film measured at T = 10.0, (b) 100.0, (c) 200.0, and (d) 300.0 K. The shape of the out-of-plane M-H curves was consistent with the ANE and AHE results shown in Figure 2(a–d). The saturation magnetization (M_s_) of Co_2_FeSi slightly increased with decreasing T. At 10 K, μ_0_M_s_ = 1.1 T, which corresponded to a magnetic moment of ~4.2 μ_B_/f.u.


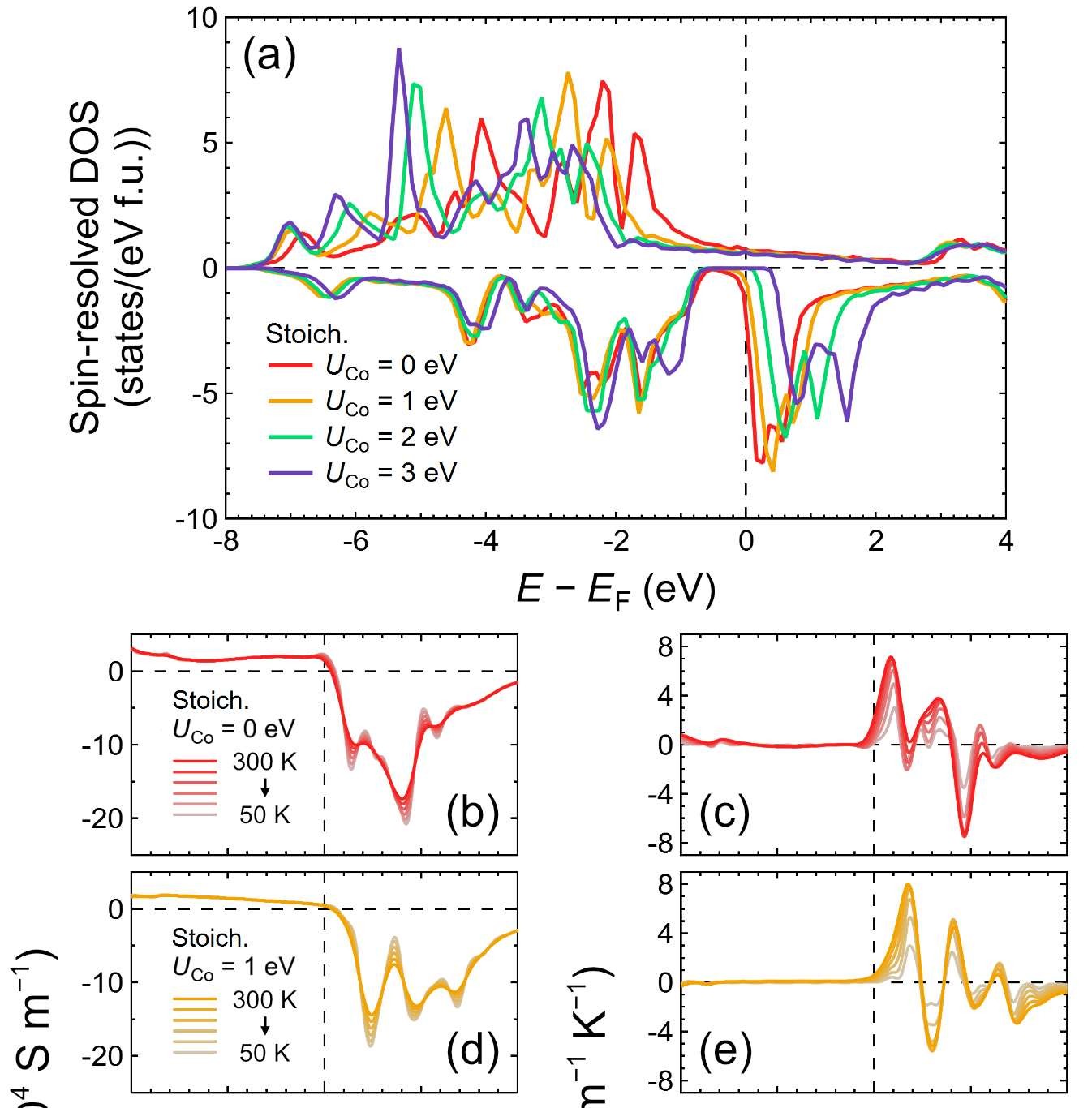

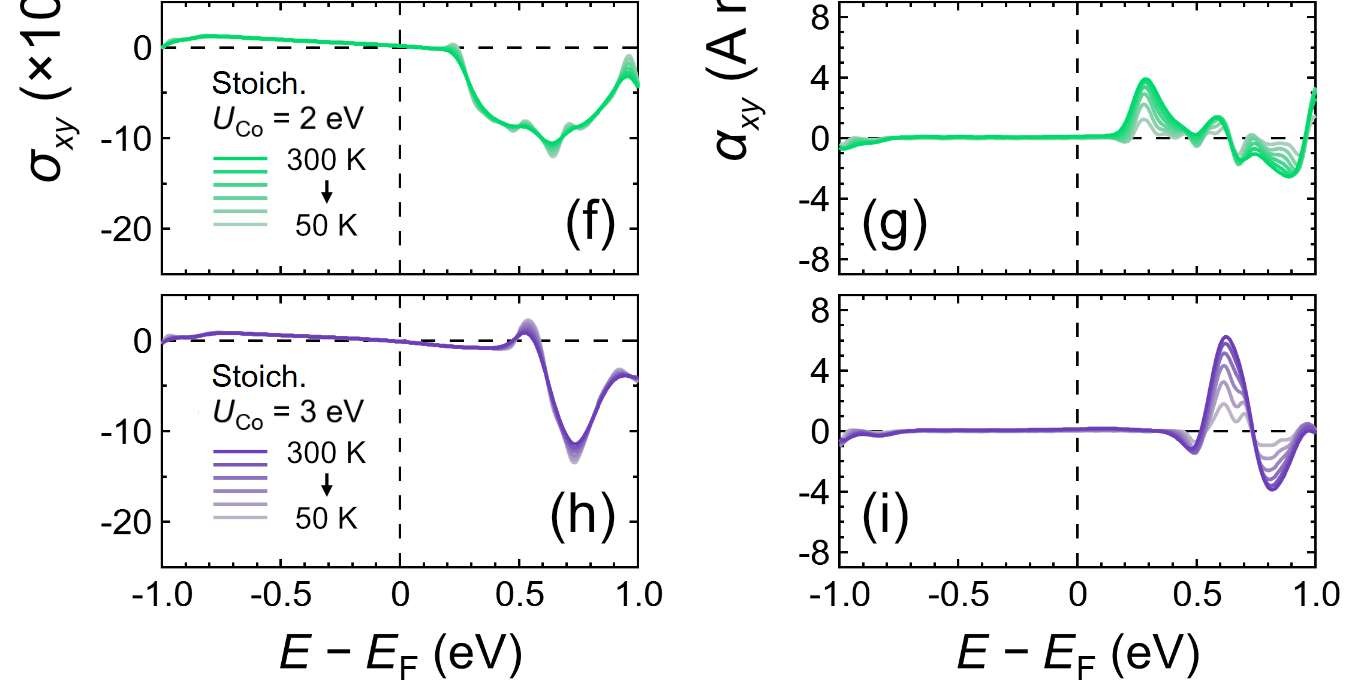


Figure S3. (a) Spin-resolved DOS values for Co_2_FeSi with stoichiometric (Stoich.) composition around the Fermi level (E_F_), with various values for the on-site Coulomb interaction at the Co site (U_Co_). (b) Calculated σ_xy_ values in the energy window of ±1 eV relative to E_F_ with U_Co_ = 0 eV, (d) U_Co_ = 1 eV, (f) U_Co_ = 2 eV, and (h) U_Co_ = 3 eV. (c) Corresponding α_xy_ values with U_Co_ = 0 eV, (e) U_Co_ = 1 eV, (g) U_Co_ = 2 eV, and (i) U_Co_ = 3 eV. The saturation of colors of the lines indicates the T values of the results, which ranged from 50 to 300 K in 50 K steps. The values of α_xy_ and σ_xy_ at E_F_ in (b)–(i) as functions of T are shown in Figure S4(a, b), respectively.


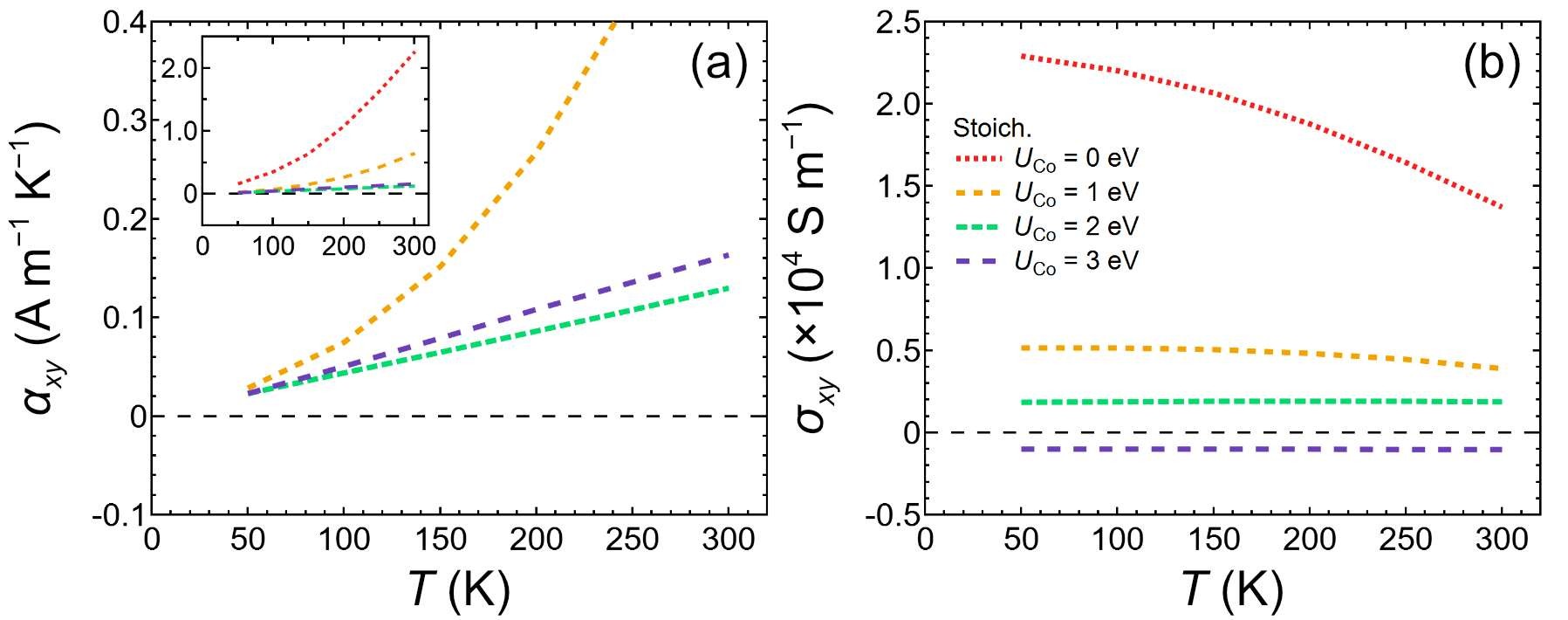


Figure S4. (a) α_xy_ and (b) σ_xy_ values at E_F_ of stoichiometric (Stoich.) Co_2_FeSi with various values for U_Co_. The inset of (a) shows the calculated results over a wider range for the y-axis. The legends in (b) also apply to (a).
